# Social support during intensive care unit stay might improve mental impairment and consequently health-related quality of life in survivors of severe acute respiratory distress syndrome

**DOI:** 10.1186/cc5070

**Published:** 2006-10-16

**Authors:** Maria Deja, Claudia Denke, Steffen Weber-Carstens, Jürgen Schröder, Christian E Pille, Frank Hokema, Konrad J Falke, Udo Kaisers

**Affiliations:** 1 Department of Anesthesiology and Intensive Care Medicine, Charité, Campus Virchow Klinikum, Universitätsmedizin Berlin, Augustenburger Platz 1, D-13353 Berlin, Germany

## Abstract

**Introduction:**

We investigated health-related quality of life (HRQoL) and persistent symptoms of post-traumatic stress disorder (PTSD) in long-term survivors of acute respiratory distress syndrome (ARDS). We wished to evaluate the influence of PTSD on HRQoL and to investigate the influence of perceived social support during intensive care unit (ICU) treatment on both PTSD symptoms and HRQoL.

**Methods:**

In ARDS patients we prospectively measured HRQoL (Medical Outcomes Study 36-Item Short Form; SF-36), symptoms of PTSD (Post-Traumatic Stress Syndrome 10-Questions Inventory; PTSS-10), perceived social support (Questionnaire for Social Support; F-Sozu) and symptoms of psychopathology (Symptom Checklist-90-R); and collected sociodemographic data including current employment status. Sixty-five (50.4%) out of 129 enrolled survivors responded, on average 57 ± 32 months after discharge from ICU. Measuring symptoms of PTSD the PTSS-10 was used to divide the ARDS patients into two subgroups ('high-scoring patients', indicating patients with an increased risk for developing PTSD, and 'low-scoring patients').

**Results:**

HRQoL was significantly reduced in all dimensions in comparison with age- and gender-adjusted healthy controls. Eighteen patients (29%) were identified as being at increased risk for PTSD. PTSD risk was significantly linked with anxiety during their ICU stay. In this group of patients there was a trend towards permanent or temporary disability, independent of the period between discharge from ICU and study entry. Perceived social support was associated with a reduction in PTSD symptoms (Pearson correlation; *p *< 0.05). *Post-hoc* test revealed a significant difference between 'high-scoring patients' and 'low-scoring patients' with respect to mental health, although they did not differ in physical dimensions.

**Conclusion:**

HRQoL was reduced in long-term survivors, and was linked with an increased risk of chronic PTSD with ensuing psychological morbidity. This was independent of physical condition and was associated with traumatic memories of anxiety during their ICU stay. Social support might improve mental health and consequently long-term outcome including employment status.

## Introduction

Health-related quality of life (HRQoL) as a state of physical, mental and social well-being is used as a measure of a patient's self-perceived outcome after critical care. There is some evidence that survivors of severe acute respiratory distress syndrome (ARDS) demonstrate significantly reduced HRQoL after discharge [1-3]. Their HRQoLs are comparable to those of patients who suffered from chronic illnesses such as congestive heart failure or stroke.

In addition, it has been reported that after admission to the intensive care unit (ICU) some patients report symptoms such as anxiety, pain and nightmares, which may develop into chronic psychiatric disorders including post-traumatic stress disorder (PTSD) and depression [4-6]. It has been demonstrated in ICU patients with ARDS and sepsis that PTSD has a serious effect on the self-perceived HRQoL [2]. PTSD follows traumatic occurrences outside the range of common human experience such as violent physical assaults, torture, accidents, rape or natural disasters and is characterized by a typical symptom pattern of intrusions, persistence of trauma, relevant stimuli avoidance, emotional numbing and physiological hyperarousal. Weinert and colleagues characterized traumatic events in the ICU setting in detail. They include hallucinations, paranoia, ICU noise, severe sleep disruption, communication difficulties and fear of disconnection from the ventilator [1].

Psychosocial counselling during stressful procedures is known to decrease the associated level of stress and improve the recovery process; however, it is not yet widely used for this purpose in ICUs. In addition to professional counselling, support and assistance from family or caregivers is receiving more attention and credence as an adjunct therapy in critically ill patients on ICUs. The prevention of psychiatric complications through the development of active coping strategies has recently become a focus of research interest [7,8].

The aim of this study was to evaluate HRQoL as a long-term outcome parameter in patients surviving severe ARDS, and to evaluate the relationship between symptoms of PTSD and HRQoL. Additionally, we investigated whether perceived social support during the ICU stay and the rehabilitation process might reduce PTSD symptoms and consequently might improve HRQoL.

## Materials and methods

This prospective controlled study was performed at a single university centre specializing in the treatment of patients with severe ARDS. Our clinical treatment algorithm included the inhalation of nitric oxide and extracorporeal membrane oxygenation [9]. The study was approved by the Institutional Review Board of our faculty, and informed consent was obtained from patients at the time that the questionnaires were sent.

### Patients

All patients were referred to our ICU from other German and European hospitals for the treatment of severe ARDS, and were admitted between 1991 and 2000 after transport by a specialized team. We started the study in 2002 and investigated only those patients who had been discharged from the ICU for more than one year because a diagnosis of chronic PTSD requires persistent symptoms. The patients were mailed six questionnaires that measured HRQoL, psychological disorders, perception of social support, and socio-demographic data. Patients were asked to recall the ICU stay and to answer each question promptly rather than after protracted consideration. If we received no reply from the patients, we attempted to contact them by phone three times. If at this stage we were still unable to contact the patient the case was counted as lost to follow-up. Data about age, sex, cause of ARDS, ICU length of stay, duration of mechanical ventilation, and admission scorings were extracted from the ICU database. To assess the severity of illness and ARDS, the Lung Injury Score and the Acute Physiology and Chronic Health Evaluation (APACHE) II scores were calculated and the length of ICU stay and duration of mechanical ventilation were shown [10,11]. To minimise potential selection bias resulting from a desire of some patients with PTSD to avoid recollection of their ICU stay as a symptom of PTSD, we evaluated characteristics of the non-participants in particular detail. A direct or collateral history of mental disease such as alcohol or drug abuse reported in direct or indirect anamnesis and lack of informed consent were exclusion criteria.

### Demographical data

We used a questionnaire to obtain demographical data about professional life, family status, educational level, and the current living situation of our patients.

### Post-traumatic stress disorder symptoms

The Post-Traumatic Stress Syndrome 10-Questions Inventory (PTSS-10) was developed to diagnose PTSD within the framework of the Diagnostic and Statistical Manual of Psychiatric Disorders (DSM-III) and includes symptoms of hyperarousal [12]. In the first part of PTSS-10, patients were asked about possible traumatic memories of their ICU stay and symptoms of severe illness such as pain, nightmares, anxiety, and respiratory distress. In part two, a ten-item self-report scale recorded the intensity of the following PTSD symptoms: sleep disturbance, nightmares, depression, hyperalertness, withdrawal, general irritability, frequent mood swings, guilt, avoidance of activities prompting the recall of possible traumatic events, and increased muscle tension. For each item a rating between 1 (never) and 7 (always) was possible, leading to a total score ranging from 10 to 70 points. A total of 70 points indicates severe PTSD symptoms. Using the German version of the two-part PTSS-10 to assess PTSD-related symptoms, Stoll and colleagues demonstrated criterion validity by receiver operating characteristic curve analysis and showed that a score of 35 points or more was a cutoff with a sensitivity of 77% and a specificity of 97.5% for the diagnosis of PTSD [13]. The ability of PTSS-10 in comparison to structured clinical interviews (SKID) to indicate patients at risk of developing PTSD has also been evaluated in patients with ARDS [3]. To investigate the hypothetical association between PTSD and HRQoL we divided the patients into two subgroups: 'high-scoring patients' with a PTSS-10 score of at least 35, and 'low-scoring patients' with a score of less than 35.

### Health-related quality of life

HRQoL was measured with the Medical Outcomes Study 36-Item Short Form (SF-36) [14]. This questionnaire includes eight scales, each reflecting a different so-called 'dimension of quality of life'. Four dimensions reflect physical health (physical component summary), namely physical function, physical role function, bodily pain and general health, and the four other dimensions reflect mental health (mental component summary), namely vitality, social function, emotional function and mental health. The total score lies between 0 and 100, with higher values indicating a more favourable quality of life. Normative data are available for a German-speaking population [15]. We matched healthy controls in terms of age and gender from the continuously updated norm database. SF-36 has previously been validated for critically ill patients [16].

### Symptoms of psychopathology

The Symptom Checklist-90-R contains 90 items and is a brief multidimensional self-report inventory containing the following dimensions: somatization, obsessive-compulsive, interpersonal sensitivity, depression, anxiety, hostility, phobic anxiety, paranoid ideation, and psychoticism [17]. These scales are summarized under three global indices: Global Severity Index, Positive Symptom Distress Index and Positive Symptom Total. For each of the 90 items a rating on a five-step Lickert scale between 0 (not at all) and 4 (extremely) is possible; data were presented with the use of *T *values (mean 50, SD 10). The Symptom Checklist-90-R was used to screen for psychological distress and multiple aspects of psychopathology in our patients with ARDS.

### Social support

The Questionnaire for Social Support assessed the perception of emotional and instrumental social support and social integration [18], and a German version is available [19]. This 22-item questionnaire measures the quality of social relationships and support. For each item a rating between 1 (low) and 5 (high) is possible. Scores for the three dimensions and a total score are calculated as means of summed scores. A rating of 5 points indicates the highest degree of social support.

### Statistical analysis

We used SPSS Software (SPSS for Windows, version 10.0; SPSS Inc., Chicago, IL, USA) for statistical analysis. The alpha level was set to the conventional 5%. Multivariate analysis of variance using the *F *statistic was used to test group (more than two) comparisons. Multivariate analysis of variance (MANOVA) was performed for HRQoL using only 'group' ('high-scoring patients', 'low-scoring patients', healthy controls) as a subject factor. Analysis of variance was also used to check a possible influence of time on the perceived outcome parameters in this long-term follow-up. The period between discharge of ICU and study was therefore examined as a covariant factor. *t *tests were applied as a *post-hoc* analysis to evaluate differences between two groups. Proportions were tested with a χ^2 ^test or a likelihood-quotient χ^2 ^test.

## Results

**Figure 1:**
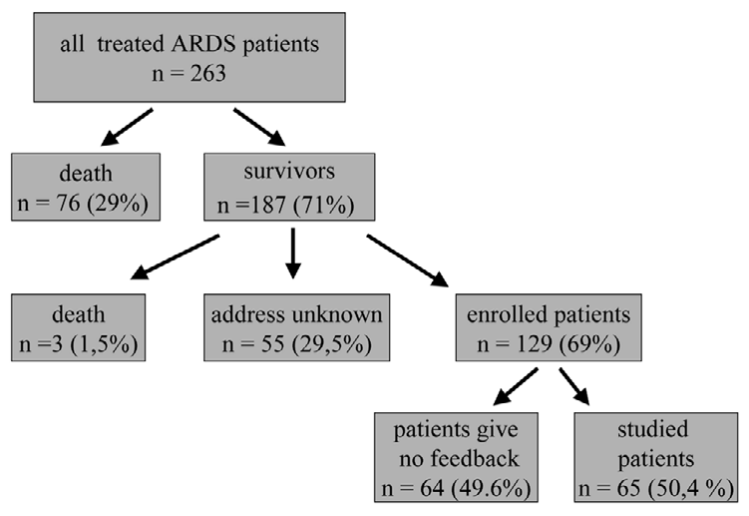
Study profile.

Between 1991 and 2000 a total of 263 patients from other hospitals were transferred to our ICU for specialized treatment for ARDS: 187 (71%) patients survived and were discharged from the ICU, and 76 (29%) died during their ICU stay (Figure 1). Of these 187 survivors the contact address was not available in 55 cases, and three patients were found to have died after discharge after follow-up with family members. Of the remaining 129 patients (69%), 64 (49.6%) did not provide feedback, and 65 (50,4%) returned the completed questionnaires and gave written informed consent (Figure 1). In the investigated patients the follow-up occurred at an average of 57 ± 32 months after discharge from the ICU (Table 1). Their demographic and clinical characteristics are presented in Table 1. There were no significant differences between investigated patients and patients who were lost to follow-up apart from the period between discharge from the ICU and entry into the study. The demographic and clinical details of these non-participating patients were as follows (mean ± SD): age, 32.9 ± 15 years; gender, 60% male; duration of mechanical ventilation, 40 ± 30 days; cause of ARDS, sepsis 10%, pneumonia 47%, multiple trauma 31%, other 14%; severity of ARDS by lung injury score, 3.2 ± 0.3; and severity of illness by APACHE II score, 17 ± 6. Only one significant difference emerged: the mean period between discharge from ICU and attempted follow-up for the purposes of the study was considerably shorter in investigated patients (57 ± 32 months) than in those who did not participate (72 ± 36 months; *t *= - 2.9; *p *< 0.0005).

### Post-traumatic stress disorder

At the time of this study, 18 patients (29%; 8 male, 10 female) were identified as being at increased risk for PTSD according to PTSS-10. Consequently we divided the entire study population into two subgroups: 'high-scoring patients' at increased risk of developing PTSD, and 'low-scoring patients'. PTSS-10 scores were significantly different between 'high-scoring patients' with increased risk for developing PTSD and 'low-scoring patients' (*t *= - 3.7; *p *< 0.0001; Table 1). Demographic data for all participating patients and the two subgroups are presented in Table 1. There were no significant differences between the subgroups in relation to age, gender, period between discharge from ICU and entry into study, duration of mechanical ventilation, cause of ARDS, the severity of ARDS as measured by means of lung injury score, or severity of illness by APACHE II score. Requirements for extracorporeal membrane oxygenation were also comparable between groups. In relation to length of stay (*t *= - 1.95; *p *< 0.056) and employment status (χ^2^(3) = 8.2; *p *< 0.084) we observed a trend towards a difference between groups. 'High-scoring patients' tended to be disabled more frequently and to stay longer on the ICU (Table 1).

A significant positive correlation between the number of traumatic memories and the severity of PTSD was revealed (Spearman *r *= 0.522; *p *< 0.0001; Table 2). In particular, a significant positive relationship between the experience of anxiety in the ICU and an increased risk of developing PTSD was demonstrated (χ^2^(1) = 7.59; *p *< 0.01; Table 3). 'High-scoring patients' at an increased risk of developing PTSD showed a tendency to recall experiences of pain more often. The whole patient group recalled nightmares or difficulties in breathing more frequently than anxiety or pain. Only experiences of anxiety differed significantly between the subgroups (Table 2).

### Health-related quality of life

**Figure 2:**
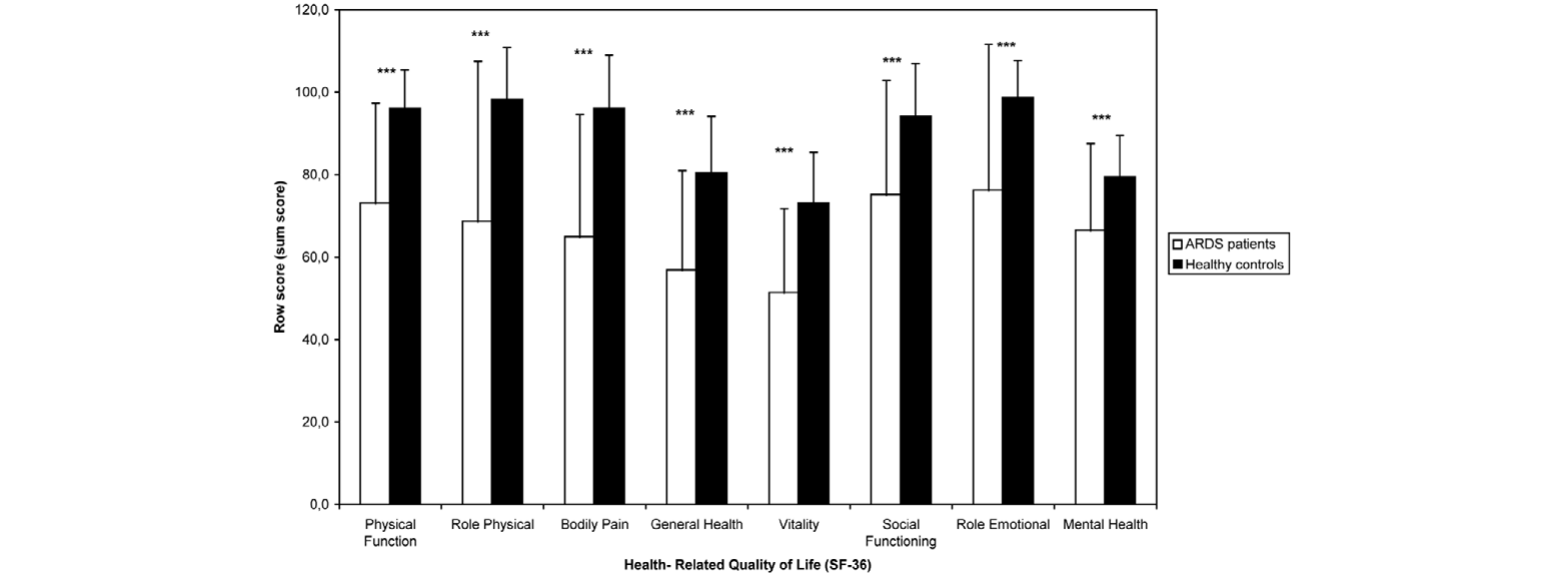
Subdimensions of health-related quality of life. Subdimensions of health-related quality of life were measured with the Medical Outcomes Study 36-Item Short Form (SF-36; physical function, physical role function, bodily pain, general health, vitality, social function, emotional function and mental health), comparing between patients with acute respiratory distress syndrome and age- and gender-matched healthy controls. Significant difference (***p < 0.0001) was calculated with *t *tests for independent samples.

**Figure 3:**
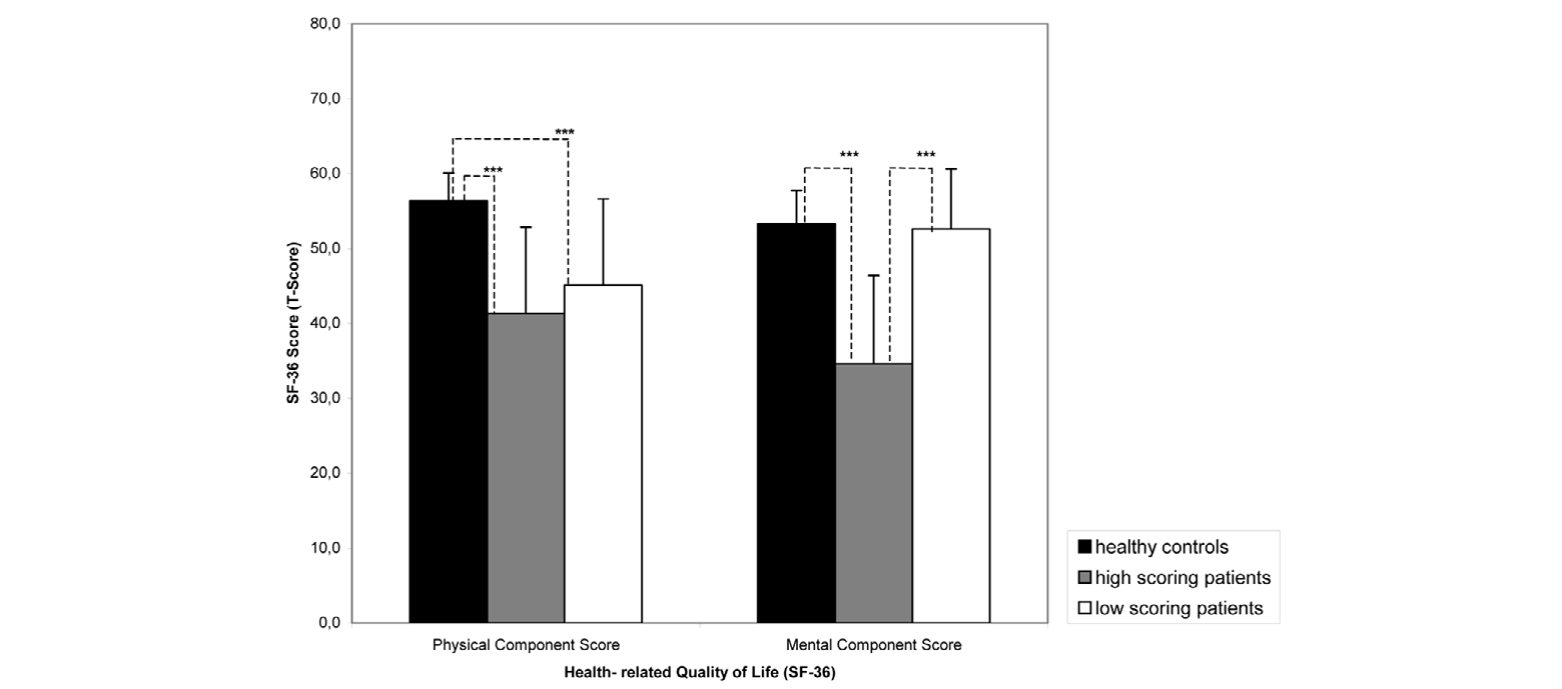
Difference in mental and physical component summary between groups. The mental and physical component summary of health-related quality of life was measured with the Medical Outcomes Study 36-Item Short Form (SF-36), comparing between 'high-scoring patients' with a Post-Traumatic Stress Syndrome 10-Questions Inventory (PTSS-10) score greater than or equal to the cutoff score of 35 points, indicating an increased risk for development of post-traumatic stress disorder, and 'low-scoring patients' with a PTSS-10 below the cutoff score, and age- and gender-matched healthy controls. Significant difference (***p < 0.0001)was calculated with analysis of variance and post-hoc *t *tests for independent samples. The broken line indicates a significant difference between groups as determined with the *t *test.

HRQoL measured by SF-36 in all patients with ARDS investigated was significantly reduced in all dimensions, physical as well as mental, in comparison with age- and gender-matched healthy controls (Figure 2). Using MANOVA we detected a significant difference between 'high-scoring patients', 'low-scoring patients' and healthy controls, and verified a significant effect between subject factor 'group' in both main dimensions (physical and mental component summary) and in all subdimensions of HRQoL. *Post-hoct *tests revealed a significant difference between 'high-scoring patients' and 'low-scoring patients' in the mental component summary as well as in all subdimensions of mental health (Table 4). In contrast, 'low-scoring patients' were not different from healthy controls in the mental component summary (Figure 3) and the subdimension mental health (Table 4). With regard to the physical component summary, *post-hoc* tests revealed a significant difference between healthy controls, and 'high-scoring patients' and 'low-scoring patients' suffering from ARDS as well as in the subdimensions physical function, physical role function, bodily pain and general health (Table 4). In contrast, there was no significant difference in the physical component score (Figure 3), bodily pain and physical role function between 'high-scoring patients' and 'low-scoring patients' (Table 4).

### Psychological impairments

Psychological problems measured by the Symptom Checklist-90-R were significantly more intense for 'high-scoring patients' than for 'low-scoring patients' in all dimensions (*t *values more than 1 SD over the mean for all scales (mean 50, SD 10); Table 5).

### Social support

**Figure 4:**
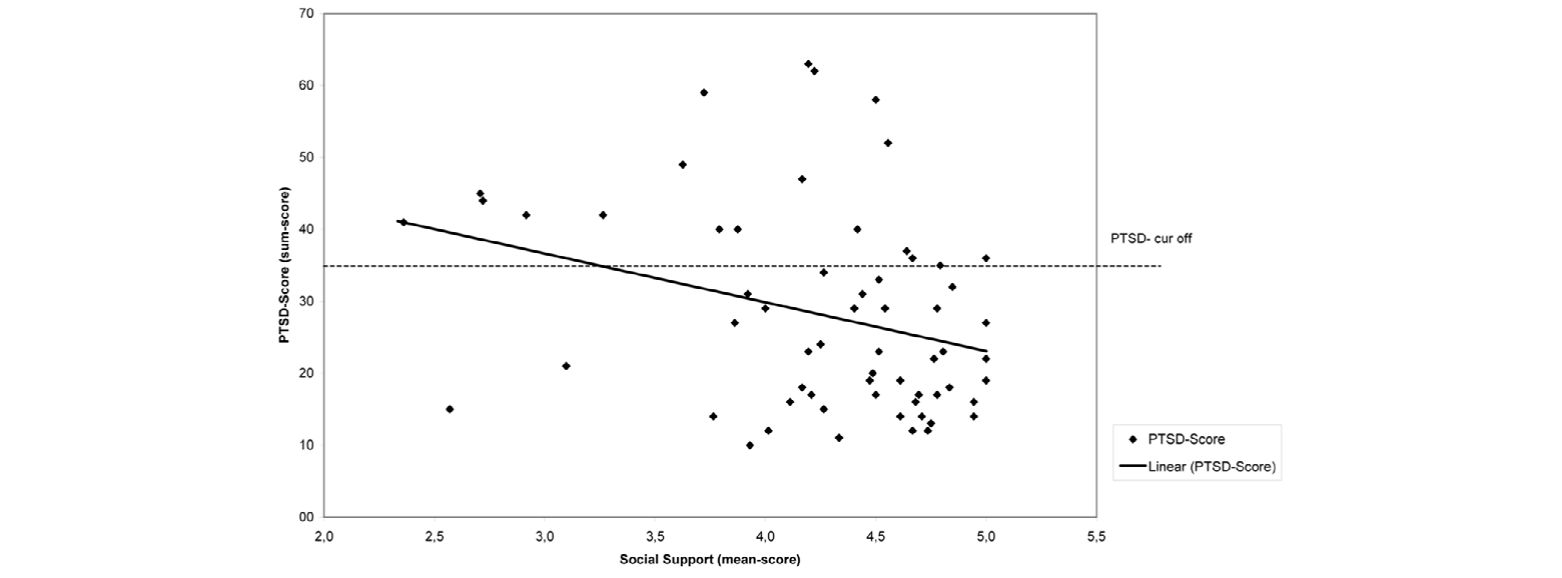
Correlation of perceived social support and posttraumatic stress. The total sum score of questionnaire F-Sozu and post-traumatic stress disorder (PTSD) score is shown. Severity of PTSD was verified with the Post-Traumatic Stress Syndrome 10-Questions Inventory (PTSS-10) score. Diagnosis of an increased risk for development of PTSD was related to a cutoff score of 35 or more in PTSS-10. The cutoff score is denoted by a broken horizontal line; social support was significantly correlated to severity of PTSD (Pearson correlation; *p *< 0.05).

Perceived social support, measured by using the total score from the Questionnaire for Social Support, was significantly higher for 'low-scoring patients' than for the 'high-scoring patients' (*t *= 2.90; *p *< 0.01). Using the F-Sozu, we demonstrated a significantly higher subdimension score for perception of emotional support (*t *= 2.24; *p *< 0.05) and social integrity for 'low-scoring patients' (*t *= 3.53; *p *< 0.01; Table 6). The perceived social support correlated negatively with the value of the PTSD score (Pearson correlation *r *= - 0.31; *p *< 0.05; Figure 4).

### Period between discharge from intensive care unit and study

Testing the period between discharge from ICU and study as a covariable, a MANOVA could not detect any influence on PTSD scores, severity of PTSD, distribution of percentages of patients suffering from recollections, psychological impairments, perceived social support and HRQoL with the exception of one subdimension of HRQoL: physical role function.

## Discussion

The aims of this study were to investigate long-term HRQoL in survivors of ARDS, to assess the influence of persistent PTSD symptoms on HRQoL, and to prove the hypothesis that perceived social support reduces the PTSD symptoms and improves HRQoL in these patients. In this study we demonstrated significantly reduced HRQoL in ARDS survivors, an association between persistence of PTSD symptoms and the reduction in HRQoL, and a possible role for social support in the prevention of PTSD. Physical impairment, as measured by the physical component score, did not seem to be responsible for the reduced HRQoLs in patients with high PTSD symptom scores (Figure 3). Furthermore, a covariance analysis indicated that physical impairment slowly but steadily improved in many patients and subsequently became less and less important. It is well known that survivors of ARDS need a long time for physical recovery. Muscle atrophy and weakness were outlined as essential prognostic factors for quality of life 1 year after surviving ARDS [20]. Using covariance analysis we observed that symptoms of mental impairment persisted much longer than symptoms of physical impairment. We were also able to show that perceived social support during the ICU stay and during the rehabilitation period was associated with a decrease in PTSD symptoms. In addition, 'high-scoring patients', indicating an increased risk of developing PTSD, more frequently applied for disability pensions.

### Trigger of post-traumatic stress disorder, the traumatic event

Psychiatric diagnosis of PTSD according to DSM-III-R criteria requires a triggering event which must be a catastrophic stressor outside the range of usual human experience. Furthermore, the stressor should be perceived as a traumatic event by nearly everyone. PTSD has a strong negative influence on QoL. This probably reflects the importance of recollection of anxiety in the development of PTSD. The lifetime prevalence for PTSD in western countries is reported with 8% within higher rates in females (10–12%) than males (5–6%) [21]. However, in some populations, the prevalence of PTSD is considerably higher, for example in ICU-survivors (28%) [2].

To measure PTSD symptoms as a result of an ICU stay we focused solely on recollections of the ICU stay. In this context, Weinert and colleagues [1] investigated patients with ARDS using interviews; they considered disease-specific questionnaires in a 'focus group' to yield more information about an ICU stay as a result of group interactions than as a result of individual memory. Their patients reported the following to be associated with anxiety: noisy ICUs, hallucinations, paranoia, fear of disconnection from the ventilator, and guilt.

### Fear of suffocation

Disconnection from the ventilator is in many cases perceived as a life-threatening situation resulting in severe emotional stress [1]. In another prospective clinical trail analysing patients 6 months after discharge from ICU, Granja and colleagues showed that in only 41% of the patients a memory of disconnection from the ventilator was associated with stress [22]; 53% of the patients recalled tracheal tube suction, and of these 81% associated the procedure with stress. In our study 'difficulty breathing' was recalled by 68% of all patients, but the frequency of these experiences did not differ between 'high-scoring patients' and 'low-scoring patients'. It seems that it is not the procedure itself but rather the individual's experience of it that determines the development of psychological sequelae of intensive care treatment.

### Nightmares

In our study 74% of all patients remembered 'nightmares', but their incidence was comparable between 'high-scoring patients' and 'low-scoring patients'. In the study of Granja and colleagues an unexpectedly low rate of 30% of all patients experienced 'nightmares', but when they did occur they had a tremendous effect on quality of life after discharge [22]. In our opinion the subjective perception of nightmares as a fearful experience is the crucial factor in the development of PTSD after treatment on intensive care wards.

### Effect of mechanical ventilation

The duration of mechanical ventilation was not associated with the severity of PTSD symptoms in our study, suggesting that mechanical ventilation itself does not affect the development of PTSD. Kress and colleagues investigated psychological effects of daily interruption of sedation [23]. Patients without daily interruption tended to recall awakening in ICU more often than those whose sedation was interrupted daily. Moreover, study patients with a daily interruption of sedation showed significantly fewer symptoms of PTSD. However, the patients did not differ in terms of HRQoL. A perception of the ICU situation that is close to reality improves the integration of treatment experiences into episodic memory, and it might prevent the formation of a memory of traumatization. Moreover, the study group of Kress and colleagues might have been too small to prove the influence of mechanical ventilation (5.6 days on average) on HRQoL. It is worth repeating that weaning strategies deploying early spontaneous breathing require appropriate strategies to avoid fear, anxiety and the feeling of helplessness.

### Strategies for prevention

Jones and colleagues described the influence of delusional memories (nightmares, dreams and hallucinations) on acute symptoms of PTSD [24]. In their study, factual memories in particular protected against the development of acute symptoms of PTSD. Even though factual memories were sometimes unpleasant, they may have helped in coping with moments of unavoidable traumatic events. Our patients benefited from social support, notably in terms of social integrity. Even though Kapfhammer and colleagues found no correlation between social support and PTSD rate, they demonstrated a reduced social function in patients with PTSD, which in turn led to diminished social activities and communication [3]. The compromise of social function in their patients may have partly induced an avoidance of social relationships, and to some extent it may have led patients to reject the social support offered to them. Correlation between social support and severity of PTSD symptoms in our patients suggested that emotional support and social integration acted as factors in preventing PTSD symptoms. Because membership of the 'high-scoring' group was related to disability pension, social assistance by family caregivers might be associated with a better social outcome in ARDS survivors.

With regard to psychosocial characteristics, it has been demonstrated that objective injury criteria are not correlated with the incidence of PTSD in trauma patients who are evaluated during the first 3 weeks after the accident, whereas pre-trauma variables such as gender and mental health, biographical risk and stressful life events associated with PTSD symptomatology are correlated [25]. The association between high social support and fewer PTSD symptoms might reflect a better social and emotional state before the ICU trauma [25]. Central factors in the development of active coping strategies and a stable mental health status to prevent traumatization are emotional support, empathy and helpful accepting behaviour of family caregivers during the life-threatening and traumatic ICU stay. Passive coping strategies, which are related to diminished social support, inhibit cognitive function and psychological recovery from a traumatic event. A meta-analysis identified a lack of social support after the trauma as one of the major risk factors for PTSD [26]. In addition, family characteristics, for example family dysfunction or instability, seem to be a risk factor for the development of PTSD symptomatology [27]. In contrast, high social support might imply good communication and a stable family network and might consequently constitute a protective factor against the development of PTSD.

There are several limitations to this study. The great variance of period after discharge in our study might be responsible for a lower feedback rate. Feedback rate and questionnaire results might have been biased by PTSD patients seeking to avoid memories of their ICU treatment. Structured interviews on discharge from the hospital might be more accurate and might encourage patients to participate in follow-up studies later in life. We chose chronic PTSD as an outcome because the diagnosis of PTSD requires a 6-month interval after the traumatic events. Fortunately, acute PTSD symptoms frequently resolve spontaneously [3]. Selection bias as a result of the avoidance of memories of the trauma might be acceptable because several patients with numerous traumatic memories responded. We showed a positive relationship between the number of traumatic memories and the value of PTSS-10, confirming the results of previous studies [2]. Numerous studies have demonstrated that both the physical and, in particular, the mental convalescence of patients with ARDS takes a long time after discharge from ICU [20,28]. In addition, non-participating patients had similar patient characteristics.

The period between discharge from ICU and entry into the study was not important for any of the investigated parameters with the exception of physical role function. This may serve as an indicator for the strength and representativeness of the study group. We wanted to investigate the long-term outcome of survivors in a specialized centre for the treatment of ARDS. The high rate of 'nightmares' in our study compared with the study of Granja and colleagues might be a result of the duration of analgosedation [22]. Our critically ill patients had a tenfold longer length of ICU stay with a correspondingly longer duration of analgosedation in than their patients. Interestingly, the length of stay was correlated with the development of PTSD. Unfortunately, we did not score acute delirium. Further studies should measure patients' experience in ICU in greater detail. We assume that assessing traumatic experiences such as delirium or fear during the weaning phase and the use of a specialized score might be of further assistance in novel strategies for the treatment of delirium associated with mechanical ventilatory support and analgosedation.

## Conclusion

We conclude that even after successful ICU treatment for ARDS in terms of physical outcome, the long-term outcome of ARDS survivors measured with HRQoL is reduced as a result of persistent PTSD symptoms. Recalled social support from family or caregivers during the ICU stay and rehabilitation has a significant effect on PTSD symptoms. Social support from family members might improve coping skills for traumatic experiences in critically ill patients. The main result of our study was that social support and its probable mental health benefits may favourably affect the long-term outcome, including the employment status, of ICU patients who recover from ARDS.

## Key messages

• In these survivors of severe ARDS, anxiety is a crucial traumatic memory during the ICU stay and is significantly linked with the risk of developing PTSD.

• Physical impairment was not responsible for reduced HRQoL in patients suffering from persistently high PTSD symptom scores (a high score indicates an increased risk of developing PTSD). Physical impairment slowly but steadily improved in many patients and subsequently became less and less important.

• Psychiatric symptoms persisted much longer than symptoms of physical impairment.

• Recall of social support during a burdensome ICU stay and rehabilitation may be positively associated with subsequent mental health, risk of PTSD and long-term outcomes including employment status.

## Abbreviations

APACHE = Acute Physiology and Chronic Health Evaluation; ARDS = acute respiratory distress syndrome; HRQoL = health-related quality of life; ICU = intensive care unit; MANOVA = multiple analysis of variance; PTSD = post-traumatic stress disorder; PTSS-10 = Post-Traumatic Stress Syndrome 10-Questions Inventory; SF-36 = Medical Outcomes Study 36-Item Short Form.

## Competing interests

The authors declare that they have no competing interests.

## Authors' contributions

MD and CD contributed equally to this study. MD made a substantial contribution to the protocol design and interpretation of data, and wrote the manuscript. CD was responsible for study concept, designed the methods, realized the statistical data analysis and made a substantial contribution to the interpretation of data. SW-C enrolled the patients and contributed substantially to the interpretation of data. JS contacted the ARDS patients, collected the questionnaire data and participated in the statistical analysis. CEP enrolled the patients, coordinated the data collection and acquired the medical data. FH helped to draft the manuscript and made substantial contributions to the discussion and interpretation of the data. KJF revised the manuscript for important intellectual content. UK contributed to the design and coordination of the study. All authors read and approved the final manuscript.

## Figures and Tables

**Table 1:** Demographic and clinical characteristics of patients studied

Characteristic	All studied patients	'High-scoring patients'	'Low-scoring patients'
Risk for development of PTSD, *n *(percentage)	65	18 (29%)	44 (71%)
PTSD score (mean ± SD)	28.1 ± 13.9	46.3 ± 8.9	20.6 ± 7.1^a^
Age, years (mean ± SD)	39 ± 15	41 ± 13	39 ± 15
Sex, *n *(male/female)	35/30	8/10	25/19
Period between discharge and study, months (mean ± SD)	57 ± 32	56 ± 33	59 ± 31
Lung Injury Score (mean ± SD)	3.2 ± 0.5	3.3 ± 0.5	3.2 ± 0.5
APACHE II score (mean ± SD)	16 ± 6	15 ± 4	16 ± 6
ICU length of stay, days (mean ± SD)	47 ± 32	59 ± 37.0	42 ± 29^b^
Duration of mechanical ventilation, days (mean ± SD)	30 ± 22	37 ± 22	27 ± 22
Cause of ARDS, *n *(percentage within group)			
Sepsis	13 (20)	5 (28)	8 (18)
Pneumonia	28 (43)	7 (39)	20 (45.5)
Multiple trauma	20 (31)	5 (28)	14 (32)
Other	4 (6)	1 (6)	2 (4,5)
ECMO, *n *(percentage within group)	7 (11)	3 (17)	4 (9)
Current status of employment, *n *(percentage within group)			
Work/training	29 (46)	7 (39)	22 (50)
Unemployed/working at home	5 (8)	1 (5.5)	4 (9)
Retired	7 (10)	1 (5.5)	6 (14)
Disabled	12 (18)	8 (44)	4 (9)^b^
Others	6 (13)	1 (5.5)	5 (11)
No data	3 (5)	-	3 (7)

In 2 patients (3%) there were no data for PTSD. PTSD, post-traumatic stress disorder; SD, standard deviation of the mean; 'high-scoring patients', patients with a Post-Traumatic Stress Syndrome 10-Questions Inventory (PTSS-10) score greater than or equal to the cutoff score of 35 points, indicating an increased risk of development of PTSD; 'low-scoring patients', patients with a PTSS-10 score below the cutoff score; APACHE, Acute Physiology and Chronic Health Evaluation; ICU, intensive care unit; ARDS, acute respiratory distress syndrome; ECMO, extracorporeal membrane oxygenation. Significance was assumed at a two-tailed *p *< 0.05. ^a^Significant difference; ^b^trend.

**Table 2:** Effect of number of traumatic memories on symptoms of post-traumatic stress disorder

Group	PTSS-10 score (mean ± SD)	Number of recollections, *n *(percentage)				
		
		0	1	2	3	4
All patients	28.1 ± 13.9	3 (5)	7 (12)	17 (29)	13 (22)	19 (32)
'High-scoring patients'	46.3 ± 8.9	0 (0)	0 (0)	3 (17)	6 (33)	9 (50)
'Low-scoring patients'	20.6 ± 7.1	3 (7)	7 (17)	14 (34)	7 (17)	10 (24)
Difference	*t *= - 3.74; *p *≤ 0.0001					

Comparison of Post-Traumatic Stress Syndrome 10-Questions Inventory (PTSS-10) score in all patients with the use of the *t *test to prove a difference between 'high-scoring patients' with a PTSS-10 score greater than or equal to the cutoff score of 35 points, indicating an increased risk for the development of post-traumatic stress disorder, and 'low-scoring patients' with a PTSS-10 score below the cutoff score. The number (percentage) of patients yielding the indicated numbers of recollection with respect to pain, difficulties in breathing, nightmares and anxiety are also shown. SD, standard deviation of the mean.

**Table 3:** Effect of several traumatic memories on post-traumatic stress disorder

Group	Traumatic memories (percentage)			
	
	Pain	Difficulties in breathing	Nightmares	Anxiety
All patients	61	68	79	63
'High-scoring patients'	78	78	89	89
'Low-scoring patients'	53	63	74	51
Difference	χ^2 ^= 3.3; *p *= 0.087^b^	n.s.	n.s.	χ^2 ^= 7.6; *p *≤ 0.008^a^

The proportion of patients recalling traumatic memories in different groups (all patients, 'high-scoring patients' with a Post-Traumatic Stress Syndrome 10-Questions Inventory (PTSS-10) score greater than or equal to the cutoff score of 35, indicating an increased risk for development of post-traumatic stress disorder, and 'low-scoring patients' with PTSS-10 score below the cutoff score). The χ^2 ^test revealed a significant difference of frequency in anxiety memories between groups. n.s., non-significant difference. ^a^Significant difference; ^b^trend.

**Table 4:** Health-related quality of life

Psychological test and group	HRQoL	ANOVA			*Post-hoc* test
			
		*F*	d.f.	*p*	
Physical component score		34.42	2;122	0.0001	^a, b^
'High-scoring patients'	41 ± 11				
'Low-scoring patients'	45 ± 12				
Healthy control	56 ± 4				
Mental component score		48.94	2;122	0.0001	^a, c^
'High-scoring patients'	37 ± 12				
'Low-scoring patients'	52 ± 8				
Healthy control	53 ± 4				
Physical function		36.81	2;122	0.0001	^a, b, c^
'High-scoring patients'	61 ± 28				
'Low-scoring patients'	78 ± 21				
Healthy control	96 ± 9				
Physical role function		17.92	2;122	0.0001	^a, b^
'High-scoring patients'	58 ± 36				
'Low-scoring patients'	72 ± 40				
Healthy control	98 ± 13				
Bodily pain		35.81	2;122	0.0001	^a, b^
'High-scoring patients'	55 ± 33				
'Low-scoring patients'	68 ± 27				
Healthy control	96 ± 13				
General health		37.52	2;122	0.0001	^a, b, c^
'High-scoring patients'	41 ± 22				
'Low-scoring patients'	62 ± 23				
Healthy control	80 ± 14				
Vitality		63.80	2;122	0.0001	^a, b, c^
'High-scoring patients'	30 ± 14				
'Low-scoring patients'	59 ± 16				
Healthy control	73 ± 13				
Social function		37.61	2;122	0.0001	^a, b, c^
'High-scoring patients'	51 ± 28				
'Low-scoring patients'	86 ± 18				
Healthy control	94 ± 13				
Emotional function		31.32	2;122	0.0001	^a, b, c^
'High-scoring patients'	49 ± 44				
'Low-scoring patients'	87 ± 25				
Healthy control	99 ± 9				
Mental health		54.11	2;122	0.0001	^a, c^
'High-scoring patients'	43 ± 17				
'Low-scoring patients'	76 ± 14				
Healthy control	79 ± 10				

Health-related quality of life (HRQoL) was measured in controls, in 'high-scoring patients' with a Post-Traumatic Stress Syndrome 10-Questions Inventory (PTSS-10) score greater than or equal to the cutoff score of 35, indicating an increased risk for development of PTSD, and in 'low-scoring patients'. HRQoL (results shown as means ± SD) was measured with the Medical Outcomes Study 36-Item Short Form (SF-36) in physical component summary (subdimensions physical function, physical role function, bodily pain and general health) and mental component summary (subdimensions vitality, social function, emotional function and mental health). The difference between groups was evaluated with an analysis of variance (ANOVA) and post-hoc *t *tests for independent samples. PTSD, post-traumatic stress disorder. ^a^Significant difference between healthy controls and 'high-scoring patients' with a PTSS-10 score greater than or equal to the cutoff score, indicating an increased risk for development of PTSD; ^b^significant difference between healthy controls and 'low-scoring patients' with a PTSS-10 score below the cutoff score; ^c^significant difference between 'high-scoring patients' and 'low-scoring patients'.

**Table 5:** Psychological impairment

Dimensions	'High-scoring patients (*n *= 18)	'Low-scoring patients' (*n *= 44)	*t *test		
			
			*t*	d.f.	*p*
Somatization	69 ± 11	56 ± 12	- 3.92	60	0.0001
Obsessive-compulsive	68 ± 10	50 ± 11	- 6.20	60	0.0001
Interpersonal sensitivity	68 ± 11	49 ± 10	- 6.63	60	0.0001
Depression	70 ± 13	49 ± 9	- 6.21	60	0.0001
Anxiety	72 ± 8	52 ± 10	- 7.12	60	0.0001
Hostility	69 ± 11	50 ± 9	- 6.94	60	0.0001
Phobic anxiety	67 ± 10	50 ± 9	- 6.28	60	0.0001
Paranoid ideation	66 ± 9	49 ± 10	- 6.30	60	0.0001
Psychoticism	70 ± 9	49 ± 9	- 8.62	60	0.0001
Global Severity Index	73 ± 8	51 ± 11	- 7.60	60	0.0001

Psychological impairment is shown on the basis of scales of the Symptom Checklist-90-R (SCL-90-R); scores are given as means ± SD. For each of the 90 items a rating on a five-step Lickert scale between 0 (not at all) and 4 (extremely) was possible. Data are presented as *T *values (mean 50; SD 10). In addition, *T *values of all patients are presented. For comparison between 'high-scoring patients' with a Post-Traumatic Stress Syndrome 10-Questions Inventory (PTSS-10) score greater than or equal to the cutoff score of 35, indicating an increased risk for development of post-traumatic stress disorder, and 'low-scoring patients' with a PTSS-10 below the cutoff score, a *t *test for independent samples was used.

**Table 6:** Perceived social support

F-Sozu dimension	All patients (*n *= 62)	'High-scoring patients' (*n *= 18)	'Low-scoring patients' (*n *= 44)	*t *test		
				
				*t*	d.f.	*p*
Total social support	4.3 ± 0.8	3.9 ± 1.0	4.4 ± 0.5	2.90	22.6	≤ 0.01
Emotional support	4.4 ± 0.8	4.2 ± 0.9	4.4 ± 0.7	2.24	20.9	≤ 0.05
Practical support	4.0 ± 0.8	3.5 ± 0.9	4.3 ± 0.6	1.88	60	≤ 0.064^a^
Social integrity	4.2 ± 0.7	3.9 ± 0.8	4.4 ± 0.5	3.53	23.4	≤ 0.005

The Questionnaire for Social Support (F-Sozu) was used to compare 'high-scoring patients' with a Post-Traumatic Stress Syndrome 10-Questions Inventory (PTSS-10) score greater than or equal to the cutoff score of 35, indicating an increased risk for development of post-traumatic stress disorder, with 'low-scoring patients' with a PTSS-10 below the cutoff score. Scores are shown as means ± SD. For each of the 22 items a rating between 1 (low support) and 5 (high support) was possible. A post-hoc *t *test was applied for independent samples. ^a^Trend.
